# Miscellanies

**Published:** 1836-07-01

**Authors:** 


### MISCELLANIES.

### On the Prone Position in Affections of the Spine, Hips, &c. By Mr. Verral.

A paper on this subject was lately read at the Westminster Medical Society, from which we shall make an extract, as it records the case in which the apparatus (very simple) was first tried.

"A young gentleman, aged 16, had for two years been afflicted with diseased spine. The complaint had come upon him during a tour in Scotland, and on his return from thence, he made a stay of some months at Hinchley, that he might be under the care of Mr. Cheshire. As he was becoming worse, he at length proceeded to London, and his friends were advised by the medical gentlemen who were there consulted, to take him to the sea-side. Lodgings were taken at Seaford, where I then resided, and in November, 1825, he was accidentally placed under my care in consequence of his having been attacked with scarlet fever. He had previously been attended occasionally by a medical friend of the family, residing some distance off; but having been thus called upon, I was of course made acquainted with the disease under which he had so long been suffering. After his recovery from scarlet fever, I therefore frequently saw him, and so great a sufferer it has rarely been my lot to behold. He had long had an abscess discharging most profusely in the right groin. There was disease and a very large posterior curvature of the lumbar vertebræ, with which the abscess no doubt communicated. He was exceedingly emaciated, and the skin so readily gave way, that every part upon which he had pressed but for a few hours had become excoriated. He had a large sore on the edge of the sacrum, another on the point of the protruded vertebræ, and another on each hip. In bed, therefore, whether lying supine or on the sides, he suffered extreme torture, alleviated but for a very few hours by the administration of the most powerful opiates. From his bed, then, he was obliged to rise, and in doing that, he daily suffered an hour of agony, which although he bore pain with much fortitude, forced tears in torrents from his eyes. When up, he sat in a chair contrived by Mr. Cheshire, with high arms and with a strap supported by an arch of steel, that came forward over his head, so that the weight of the upper part of the body might be taken off from the diseased portion of the spine. But his elbows had been wrung by leaning on the arms of the chair, and his chin was completely excoriated by the frequent use of the strap. For ease, therefore, he had recourse to his crutches, upon which he sometimes stood for two or three hours at a time; but at last excoriation took place in each axilla, and thus every change of position afforded him but a change of torment. Having one day witnessed his sufferings, I went away, considering whether it might not be possible to place him in some other position, which would afford him sometimes an hour or two of respite from the terrible tortures which, in addition to the disease, were evidently hurrying him to the grave. I thought of the haymakers and harvesters, and the shepherds, whom I had often seen sleeping on their faces; and although Horace, I believe, tells us that 'Malus pastor dormit supinus,' I concluded that, as it is *Malus* pastor who so sleeps, I would even venture to send my young friend to sleep in the PRONE POSITION. By way of trial I threw myself at length on the sofa, but I found that a regular platform gave an uneasy stretching to the body, which I felt assured he would not be able to bear. After many trials, I found that a slight angle in the platform, corresponding with the bend of the hips, rendered the position extremely easy. Upon this hint I acted, and having also contrived an easy mode of laying the patient down upon the board, I had it one day taken to the house, and he was immediately placed upon it. In three minutes he was asleep, and continued so for three hours. When he awoke, seeing me sitting beside him, he exclaimed that he had to thank me for the sweetest sleep which he had enjoyed for many months. That night and the two or three following ones he was removed from the couch to his bed, but after that he resolved to try a night upon the couch, and succeeded so well that from that day, which was the 22d of December, until he died on the 28th of the following August, he never but once left it, excepting for the purpose of that daily change which the great discharge of the abscess rendered imperatively necessary. On this account it was that I was obliged to contrive such additions to the original platform as were required to make it a permanent instead of an occasional place of rest, and at the same time to enable him to be moved off and on with facility. This was effected, as I shall shew you, in so simple and yet in so effectual a manner, that, whether laying on the couch or in being raised so that he might be changed, and the parts cleansed and dressed, he never complained of the slightest degree of pain. He rested without an opiate, amused himself during the day in reading, writing, and drawing, all which he could accomplish without the slightest movement of the body; and, to add to our surprise, although in no other position was he able to lie, even for a few hours, without wringing, he lay in this manner for more than eight months, and had not even in any part the slightest reddening or excoriation. He recruited so much at first, in consequence of the ease and rest he was experiencing, that for a short time we had almost a hope of his ultimate recovery. Fresh abscesses, however, formed; openings took place in the other groin, and in the thighs, and a violent hæmorrhage at length coming on, he expired, as he had prayed to do, on his 'dear couch,' as he called it, without feeling a pang, and without uttering a groan. In this, and in one other case, we suffered some inconvenience from an œdematous swelling of the legs, but it never became of any serious importance. It was, however, the cause of his trying a change from the couch to his bed on one night, which was, as I stated, the only time that he attempted that change after he had made the couch a permanent resting place. This was done at the request of the medical friend I have alluded to, but the experiment caused him so much pain, and before I saw him, which was not till the next day at noon, had again so much excoriated the angle of the distorted spine, that it was never again repeated. During the last week or two he had become so weak as scarcely to bear the upright position, in which he was placed for a few minutes to be washed and changed, and I was thinking, at the time of his death, of contriving to fix on the couch a corresponding board, which, being brought over his back, he might have been rolled over and back again without the slightest personal exertion. This, I think, might be easily effected, but I have never since had a case that has required it.

On this case I have few observations to make. By no mode of treatment do I believe it capable of being cured, at the period when it fell under my observation. But I also believe that no position in which the patient could have been placed, would have afforded him so good a chance of recovery; and if the disease had not already committed such dreadful ravages, if the bones had not become altogether carious, and if the constitution had not been worn down by long suffering, and profuse discharge, then do I almost believe that the perfect ease which the couch afforded him, the free exit that it gave to the pus, and the great freedom from motion which was so long allowed to the diseased parts, would have insured him an ultimate cure. From the observations which I made on the above case, I may with confidence assert, that in all cases of psoas abscess, in which the matter is pointing, or has made its way anteriorly, the prone position is the one in which the patient ought to be laid. Indeed, in the early stage of that disease, while it may be hoped that active remedial measures, such as leeches, issues, blisters, friction, &c. might be the means of preventing the formation of matter, it is clear that in no other position can the patient be so advantageously placed, as well on account of the perfect quiet that it affords, as of the extraordinary facility which it gives for the application of the various remedies. In the incipient stage of disease of the vertebral column, the prone position is still more imperatively demanded; and in that case, as well as in inflammation or injuries of the spinal marrow, or of its investing membranes, the long-continued and most perfect quietude in which the patient may remain, is assuredly a most important recommendation. Where there is no purulent discharge, to render a change of linen frequently necessary, there need be no limit to that state of quiet, but what the commonest cleanliness would impose, as the food is taken, the evacuations passed, and all the offices of life carried on without the slightest movement of the body."*

The apparatus is abundantly simple —being, in fact, only a board about three feet broad, with a hinge in the middle, or nearly in the middle, capable of being bent to any desired angle, and on which a thin mattrass is spread. There is an aperture for the feet, to give them some support when necessary, and the knees may be bent by placing a pillow under the ankles. We are quite unable to speak to the utility of this pronation-bed from personal experience. To many members of the Westminster Medical Society, it appeared to promise advantages, especially in ulcers and sloughings about the back and nates, as well as in injuries of the spine; but future observation alone can determine these points, inventors being generally found to be too partial to the creatures of their own creation.

There is, however, one potent drawback on Mr. Verral's proposal—namely, that it will be opposed by the whole of the ultra or tory aristocracy of the profession. They will, doubtless, consider this as one of those topsy-turvy projects for turning every thing upside down, and undermining the foundations of our VENERABLE INSTITUTIONS. The medical aristocracy have long *preached* the doctrine of *supination* to the inferior classes of their order; but they took good care never to *practise* it. Mr. Verral may rest assured that they will discourage every *movement* from the supine posture—even that of the inclined

---

* See Dr. Ryan's Journal for May 28th, 1836.

plane—as tending to that much dreaded *equality* which sounds so harshly in aristocratic ears.

---

### BLEEDING IN THE COLD STAGE OF INTERMITTENTS.

(India Journal.)

We perceive that Dr. Mackintosh's plan is gaining ground in India, where an ample field is open for experiment.

" The novelty of this practice, and the dangerous consequences supposed to result from it, have deterred many from pursuing this mode of treatment. It is but natural that we should receive with distrust and adopt with caution, a doctrine, which seems so totally at variance with all our preconceived notions of the nature and treatment of this disease. The employment of V. S. is one of the newest lights, which has dawned on us, and is I believe a coruscation from the luminous and inventive imagination of Dr. Mackintosh of Edinburgh, assisted by an unwearied assiduity, and perseverance, in every object, connected with the profession; to this gentleman we are indebted, for a safe and most useful prophylactic, by the judicious administration of which, this most troublesome disease is not only speedily cured, but the chronic visceral derangements, so frequently the sequelæ of its invasion, are in a great measure, if not wholly, obviated.

The practice is I believe far from being universal, in this country, and I am aware that Medical men in general, are averse to it, particularly among the natives; I confess that at first I felt some difficulty in overcoming my own prejudices, and with a wholesome dread of fatal syncope, debility, natural want of stamina, I with a trembling hand, resolved to try its effects, on an emaciated *kidmutgar,* who was ill for six days previously. Having directed him to come to me when his cold fit was about to commence, I took from his arm six ozs. of blood, when he became faint, and continued in that state for nearly half an hour; giving him every now and then a little *ammonia,* he soon called and walked home, without the assistance of two friends who had accompanied him to my house. The fever was of the quotidian type, I gave *two* grains of quinine to take the following morning, he has never had since a return of the symptoms, and enjoys his usual health. My next patient was a prisoner in the jail, a stout robust fellow, attacked with fever of the tertian type, had one paroxysm, the hot fit extremely severe, violent headache, skin hot and dry, the intermission perfect—has had no medicine; ordered him to be bled, when the next cold fit had fully commenced—the result was as I anticipated, the paroxysm was instantly arrested, the hot and sweating stages scarcely observable, and no return of fever; ordering him some purgative medicine, he was discharged —four days subsequently, he got intoxicated, which brought on a return of his former symptoms; he was, by his own particular request, treated as before, and with the same agreeable result; the quantity of blood taken in the first instance amounted to 12 oz. in the second to 10 oz. A servant of my own was the next patient on whom I tried its effects, his fever was of the quotidian form; I bled him when the cold stage had fairly set in, after which he had no regular return of fever, except that for two days after, a slight disposition to recurrence of the symptoms manifested itself, which yielded readily to small doses of quinine: except a purgative, he took no other medicine, and has since continued well. It would be useless to enumerate the many examples I have had of the efficacy of this plan. The two last months have afforded me many opportunities of putting it to the test of experience, I can therefore with confidence recommend V. S. as an invaluable remedy in this disease.

There are, however, some cases where it may be injudicious and even dangerous to adopt it, where in old exsanguineous subjects great debility and a complication of other diseases co-exist, but these are, I apprehend, few. In a case which I had lately under my care, in which the propriety of V. S. appeared questionable, I witnessed the best effects from the topical application of leeches.

An old servant, labouring under chronic enlargement of the liver, and an enormous spleen, has had tertian fever for the last sixteen months, for which quinine and various remedies have been taken in vain, applied to me lately; on examination I found that he suffered little from the diseased liver, but complained a good deal of the spleen, which became extremely painful during the fever, particularly in the cold stage: I directed the application of six leeches for three successive periods with a calomel purge; from this mode of treatment he derived the greatest benefit; pain and tenderness of the spleen much diminished, and a postponement or irregularity of the paroxysms, with a sensible decrease in severity—the subsequent exhibition of quinine, which before proved useless, now manifested its usual powers as a Febrifuge, and the poor man continues free from fever, his other complaints nearly in statu quo.

In this instance, it is, I think, clear that fever was kept up partly from the influence of habit, but in much greater degree, from the diseased spleen being unable with impunity to bear the increased influx of blood, which is directed in an especial manner, to this organ in intermittent fever.

I abstain from offering any observation as to the 'quo modo' of its action, this being a subject which requires, for its proper consideration, more time and talent than I can bestow; I should hope that some one more competent for the task would undertake it, content to add my humble efforts to that of others in recording the advantages to be derived, from the employment of a remedy at once simple and efficacious.

I cannot conclude without observing, that I feel myself indebted to Mr. Twining for having drawn my attention, and, I believe, that of the profession in this country, to this subject—a circumstance which, in itself, entitles the practice to favorable consideration, coming forth, stamped with the authority of one, whose zeal, in scientific pursuits, is always so conspicuous, and whose unbounded experience and sound judgment, so fully qualify him to form a correct estimate of its value.

T. C."

### Dublin Medical Journal.

We have great pleasure in copying the following paragraph from the last number of our Dublin contemporary. We need hardly say that it is *satis superque.*

" *The Medico-Chirurgical Review.*—In the last number of the Medico-Chirurgical Review, we observe that its Editors have expressed their disapprobation of an article in our Journal for March, in which we alluded to those temptations incident to the conducting of a Medical Journal in a metropolis; and that they have supposed we were preferring a charge of venality against them.

We assure the Editors of the Medico-Chirurgical Review, that these observations were not intended to bear upon them. Even if the charge of venality were applicable, we, at least, have received no cause for preferring it. But we make no such accusation, and we regret that an observation of a general tendency should be so misconstrued, and made the grounds of personal allusion, always a useless as well as unpleasing mode of argument. Let us in conclusion express our sincere hope, that that harmony so essential to the progress of truth may be restored, and that both works having for their object the furtherance of science, will continue in the mutual good understanding which has heretofore existed between them."

---

### Reporting from Medical Societies.

A sharp conflict lately took place in the Medical Society of Calcutta, respecting the propriety or impropriety of having correct and full reports (by a professed reporter) taken of the Society's proceedings, in order that they might be published in the India Journal of Medical Science. Mr. Corbyn, the Editor of the journal argued for the necessity of an authentic report. Mr. Hutchinson, the secretary, spoke against it—on the ground that it would injure the sale of the Society's Transactions. It appears to us that neither side took a just view of the case. Mr. Corbyn ought not to have insisted on full reports, not only of the discussions, but of the papers read in the Society—for undoubtedly such procedure would destroy the sale of the Society's Transactions. On the other hand, the Society has no property in the VIVA VOCE discussions. Such discussions are not published in the Transactions, and are lost to all except the few members actually present. The discussions, therefore, ought, on every principle of liberality and utility, to be published for the benefit of the whole profession. Authentic and unprejudiced reports, too, are of great advantage—as garbled and partial ones are prejudicial. Even with all the inconvenience of incompetent reporters—sometimes imbued with party spirit or personal hostility—the system of reporting has been beneficial to the public: Mr. Bramley, one of the disputants, observes—

"He had but to look to some of the leading medical societies in England, where the system of reporting was permitted, or even encouraged, to satisfy him that, by the introduction of a similar system here, they might get on a great deal better. He believed that the Westminster Medical Society owed its present high reputation to the reporting system."

On going to ballot, however, the proposition of Mr. Corbyn was lost by a majority of ONE! We hope that, ere long, the majority will be on the liberal side, and that the *discussions*, which are no man's property, and which may be useful to all, will be reported by a regular and impartial hand. This, indeed, is a desideratum in all societies, where the most ludicrous mistakes and important errors are at every meeting reported, from inability to take notes in short-hand.

---

### SIR WILLIAM BLIZZARD.

A MEMOIR of this venerable member of our profession has been published by Mr. Cooke, having been first read before the HUNTERIAN SOCIETY. If the intellectual powers of our departed brother had been equal to his physical stamina and tenacity of life, NINETY-FOUR YEARS of existence—with all the advantages of being a public character, and with a large hospital for the theatre of his exertions, would have furnished a far worse biographer than Mr. Cooke, with ample materials for a different kind of volume to that which lies before us—containing 67 pages!—The man who could amputate, with steady hand, at the age of 84, must have been endowed, naturally, with a firm nervous system. His retentive memory was still more remarkable than the imperturbability of his nerves. At the age of 93, he was able, without any preparation, and without having read the poem for forty or fifty years before, to repeat the whole of "Gray's Elegy in a Church-yard," almost without hesitation! If, therefore, the powers of perception, reflection, and discrimination, had borne any kind of proportion to the power of memory, the deceased might, even with very moderate application, have left behind him, for the benefit of posterity, a mass of practical facts of incalculable value to his brethren, and which would have transmitted his name to remote ages, as a benefactor to the human race! But what do we find, as the result of this Herculean *physique*, and unparaleled tenacity of impressions on the sensorium? Nothing—or next to nothing!! Three or four insulated communications (of little value) to periodical journals or transactions! This could not be attributed to indolence, for few were more active than the deceased. It must, therefore, be placed to the account, either of defective talent, or deficient zeal for the promotion of that science in which he was embarked for the greater part of a century. His biographer affirms that he was *most zealous*. The other alternative is, that he was a man of very moderate talent. We firmly believe that this was the case.

The great object of biography is, or ought to be—BENEFIT TO THE SURVIVORS. We would ask Mr. Cooke, in a line from that beautiful Elegy which the deceased retained so strongly in his memory—

> " Can friendship soothe the dull cold ear of [death ?"

Certainly not. We ought, therefore, to stimulate the living to avoid the defects of the dead, as well as to emulate his virtues. Admitting, as we do, that the deceased was a man of no bright or intellectual capacity ; yet, the length of his life, and the retention of his memory, gave him ample means of accumulating FACTS, which his public and private practice furnished him with in abundance, and which it was his duty to record, as a compensation for the benefits which he enjoyed. The medical man, of extensive experience, who dies without recording the more important points of that experience, for the good of his successors, is not a whit less reprehensible than the father who lives up to the whole of his income, and leaves a family without provision ! In short, he is a *post obit* bankrupt in science, who draws all he can from others, during life, but leaves nothing for his successors to help them on their way through the difficulties and labyrinths of their profession !

In these reflections, we merely take the example before us as a text, and not with any personal motive for recrimination. We never once came in contact with the deceased, or ever had communication with him, direct or indirect. But the biography under our notice has impressed us with the conviction that *negative merits* little deserve the eulogy of friends, though they may disarm the censure of enemies. We thank Mr. Cooke, however, for the biography of a surgeon, who attained the age of 94 years, without materially improving medical science, though educated under a HUNTER and a POTT, and the preceptor of an Abernethy.

In medical politics, Sir William was a BURDETT, in the beginning—and, alas! a BURDETT at the end! A *liberal* when his faculties were opening, and at their prime—stationary, if not retrograde, in his principles, when age had chilled the generous impulses of youth, and the EBB OF LIFE communicated its Lethean retrocession to the feeble and backward current of intellect! Yes! A Blizzard and a Burdett hold out a lesson to frail HUMANITY—mortifying indeed, but pregnant with instruction. It teaches us that those, who find themselves incapable of marching in the steps of improvement, wherein they trode in youth and manhood, should leave the path entirely, and not retard the progress of others. The majestic oak, that embellishes the avenue leading to the stately edifice, may fall in the wrong direction, when old and decayed, and thus obstruct the road which it formerly adorned and protected.

So much for the public life—which is public property—of the deceased. In private life, and in the domestic circles, we have always understood that Sir William bore a most amiable character, religious, moral, and humane.

Long as was his course on this sublunary stage, it seems to have been chequered with few or no incidents worth recording. He may be said to have lived and died within the sound of Bow-bells! Once indeed, and at the age of 84, he quitted modern Babylon, and penetrated as far as modern Athens. The contrast between that romantic city and the environs of Whitechapel and the Minories, must have astonished the old gentleman, if his sight enabled him to compare the localities ! " The journey was completed in four days ; and he commenced travelling every morning at 5 o'clock. *The only objects of attraction on the road were the hospitals of the principal towns through which he passed.*"

" The only severe indisposition he ever experienced before his last illness, (except during a single night rendered memorable by a circumstance which will be mentioned in the sequel,) occurred after a long and tedious dissection of a fine muscular subject, *in which he undertook to measure every muscle in the body.* He worked almost incessantly till its completion, frequently continuing over the subject both night and day. These exertions were followed by an attack of typhus, which very nearly brought him to the grave."

We should think that such a task was about as useful as to count every muscular fibre in the multifidus spinæ.

There is no mention made of Sir William's having ever entered into the holy state of matrimony, or of having left any family. We wonder at this. In an Appendix, some specimens of his early *political* writings, under the signature "CURTIUS," are given. They would now be characterized as not merely radical, but *revolutionary*. Sir William was no advocate for the divine rights of kings. He distinctly states—"*Reges ex nobis—non nos a Regibus.*" This means, if it mean any thing, that kings should spring from the people! Such a sentiment, however, emanated from the deceased, some 60 years since. Suppose a deputation from the *members* of the College of Surgeons had advanced to the Hall, where Sir William sat as president of the Council, 60 years after the above enunciation, with a banner, on which was inscribed—

"CONSILIUM EX NOBIS—NON NOS EX CONSILIO."

What would the sturdy KNIGHT have done? We imagine that the ponderous mace would have quickly come in contact with the standard-bearer's head, and that Sir William would have exclaimed, with stentorian voice, caitif members!

Consilium pro vobis!!
No, no.
Vos non vobis—aratra fertis BOVES!

Sir William, at the period in question, was a RADICAL REFORMER. He railed against taxes that were levied on the people to support a luxurious court, and a dominant oligarchy! One of his remedies for these and other evils, will sound rather curious in modern ears.

"An exertion of the king's prerogative, in the dissolution of Parliament, would be the means of removing *all the grievances* of Englishmen at this day."

Well! Sir William lived to see such a prerogative exerted; but the consequences were not quite what the worthy Knight expected!

Nevertheless we will do our venerable departed brother the justice to say that, although in his riper years he endeavoured to put a drag on the wheels of reform, yet he advocated many good and useful principles. Among these was, the diffusion of general instruction among the people at large. For this single advocacy we would forgive him all his sins, whether of omission or commission. But he did many other good things. He contributed mainly to the support and better regulation of the LONDON HOSPITAL, which was his favorite hobby, and the scene of his most useful labours. His excentricities were considerable, as all his contemporaries well know. He was not only a politician, but a POET, on a small scale. Most of his effusions, in this line, remain, we believe, in manuscript. His biographer has adduced a few specimens. From the last which he wrote, in March 1835, four or five months after having a cataract extracted by Mr. Lawrence, we shall extract a passage.

"HUSH! blessed Impress! sight suddenly restored!
Prostrate, I breathe thanksgiving, gracious Lord!
The face of nature, sun-gilt world, to see,
Revives each fibre's sense of function free.
But I will not the loss of sight deplore;
Its restoration animates the more.
The series of impressions on the sight,
And, in the scale of wonder and delight,
Are forms which have an organized frame,
Or vegetate, or have a sentient claim.
The human features, with mysterious sway,
Benevolence, and bravery, portray.
Yet, oft would sorrow mingle with a smile,
And sympathy, as oft, its pangs beguile.
Should *Herschel* farther move sight's marv'lous sphere,
Bigots may bode a solemn era near.
The mighty universe is the same still:—
Learn, and obey, th' adored Maker's Will!"

Considering that these lines were written in the 94th year of his age, and only a few months before death, they are by no means despicable—and, at all events, ought not to be subjected to criticism. He departed this life on the 28th of August, 1835, having attended the Court of Examiners in Lincoln's-Inn-Fields, only one week before his decease!

The estimate which we have formed of the character of our patriarchal brother, now gathered to his forefathers, is this:—his physical organization evinced a singular tenacity of life. His intellectual powers were below par, *on the whole;* though some of them, for example MEMORY, were remarkably strong.

Does this inequality prove the truth of Pope's celebrated sentiment?

> "Thus in the soul where MEMORY prevails,
> The solid power of UNDERSTANDING fails—
> Where beams of bright *imagination* play,
> The *memory's* soft figures melt away."

Be this as it may, if his intellectual powers were comparatively weak, his sense of morality, justice, and integrity were strong. Of a man's religious sentiments, it is for his Creator, and not the creature to judge. Outward professions *ought* to go for little in this world, where hypocrisy too often usurps the garb of sanctity. We can only estimate a man by his actions, and not by his thoughts. We have no reason to suspect the deceased of insincerity; and therefore we hope and *believe* that he is gone to that tribunal (before which we must all appear) with a conscience void of guilt, to receive the reward of a long, useful, and honorable life!

---

### MESENTERIC DISEASE—PERITONEAL ACCRETION. By W. A. GREEN.

(India Journal of Medical Science.)

The two following cases are somewhat similar in the train of symptoms and in the results of examination after death. The symptoms common to both were an almost daily accession of fever, or at any rate of increase of pulse, sometimes ushered in with violent shivering; the absence of pain on pressing the abdomen; extreme debility; and gradual wasting of the flesh. I did not anticipate finding Mesenteric disease, after death. In the first case Dysentery was supposed to be the influential disease, but the bowels did not exhibit signs of violent disease after death. In the second case the affection of the heart was not the main disease, but part and parcel of a most formidable mass of morbid structure, and yet in either case how few the diagnostic signs!

1*st Case.* K.B. a prisoner, aged 30, was admitted into Hospital, July 23, 1833, with dysentery which lasted more or less until December. During this period he recovered from the dysentery, and it relapsed several times, a little slimy matter was almost always to be seen in his evacuations. In December he became much more feeble, anasarcous swellings appeared generally over the body, the pulse in the morning was very feeble, fever came on in the afternoon with slight shivering and went off at night with gentle perspiration, the tongue moist and pallid, the evacuations of a pale colour. Bark was given in the absence of the fever, combined with chalk mixture when slime appeared in the stools; castor oil occasionally; antimony during the attacks of fever, and a mild diuretic.

*January.* During this month the fever diminished, but there was increase of pulse daily in the afternoon, lasting for several hours. *January* 25*th.* Fever returned ushered in with severe shivering and closing with perspiration—no slime in stools.

*February.* The debility now became extreme; carbonate of ammonia and wine were added to the bark given, the dysenteric affection came on again and was relieved, and during the latter half of the month he remained free from fever or increase of pulse in the afternoon, his evacuations feculent.

*March.* For the first half of the month he remained much the same—on the 19th he again passed slimy stools and the pulse quickened towards evening. The debility increased, he gradually sunk, and died on the 25th. The mouth had been affected for the relief of the dysentery; and blue pill, calomel, and colocynth had been occasionally given, to improve the secretions, in the course of the disease.

*Post Mortem examination.—Head.* About an ounce of fluid in the bag of the arachnoid—the vessels on the surface of the brain rather vascular.

*Chest.* The lungs were studded with recent tubercles—in the left bag of the Pleura one pint of fluid, in the right four ounces. The heart smaller than usual by one third.

*Abdomen.* The whole surface of the Peritoneum lining the abomen, and the mesentery, studded with minute seed-like depositions of lymph. The external surface of the liver covered with the same—the bloodvessels of the mesentery distended with blood and of a purple colour. The glands of the mesentery very much enlarged, some as large as unbroken almonds, upon dividing them the substance of the glands was found vascular, and in their centre a quantity of pus—the fatty appendages of the large intestines thickened and increased in size, and containing pus within their structure. There were several smooth apparently healed ulcers, and one fresh ulcer, on the mucous membrane of the large intestines.

2*d Case*. G. S. prisoner, aged 35, was admitted into Hospital, April 27, 1834. He was feeble, with a small quick pulse, rather hot, dry skin, and furred tongue, bowels in good order, a dull heavy expression of countenance, and sallow complexion, he had no cough, no uneasiness on pressing the abdomen. The symptoms remained thus. *May* 22*d*. Upon applying the ear over the heart greatly increased sound was heard beneath the sternum, and on the right side of the organ extending to beneath the right clavicle, and over the back. I considered that the disease was, enlarged right side of the heart. From about this time he had a daily attack of fever, coming on with slight shivering, sometimes in the morning, or in the afternoon, or at night and subsiding after a few hours with perspiration, during the intervals of the fever he expressed himself to be easy, his pulse which was at first small, quick and regular, latterly became feeble, he could lie in any position without distress, breathing easily, no pain in chest nor in abdomen, the respiration was heard distinctly by the ear applied to the chest, the evacuations healthy; the tongue, at first moist with a central white fur, latterly became red and dry, the skin dry. The symptoms continued, with scarcely a change, to the day of his death, there was the daily attack of fever; increasing debility, and gradual wasting of the body, with anasarcous swellings of the legs towards the close of the scene. On the 10th of July the surface became chilled, he lay during the day in an unconscious state, and died that evening. The morbidly increased sound of the heart continued violent to the last, the bowels were a little disordered for a day or two previous to death. He was treated at first with tartar emetic, salines and purgatives; subsequently for the supposed disease of heart, leeches were applied to the chest, bark and camphor, and ammonia and T: Valerian. Am. were given, and T. Digitalis and Vin. Antim. during the fever; latterly 5 grs. of Plummer's Pills every night.

*Post Mortem examination, ten hours after death.—Head.* Minute ramifications of vessels on the surface of the brain highly injected, about 2 oz. of fluid altogether within the arachnoid and ventricles. The veins of the choroid plexus congested—the brain at its base, particularly that part beneath the lateral ventricles, very soft. The brain generally somewhat softened.

*Chest.* On left side partial adhesion between the two Pleuræ, 3 or 4 oz. of fluid on this side, the lungs of both sides here and there tuberculated, the apex of the right lung somewhat consolidated—both lungs posteriorly gorged with blood, of a dark purple colour, and emitting frothy mucus when cut. The Bronchiæ and trachea filled with frothy mucus. In Pericardium 1½ oz. of fluid. Heart very small, right ventricle of half the common size, and two or three lines only thick. The pulmonary gland being under the aorta, right pulmonary artery, and ascending vena cava united into a mass, firm and cheese-like when cut.

*Abdomen.* The walls of the abdomen, and omentum, intestines, liver and spleen agglutinated together. The whole surface of the peritoneum, throughout the several viscera, the mesentery, &c. covered with small granules of lymph giving it a nutmeg-grater appearance, its surface of a deep red colour, purple congested veins ramifying over it, these granules between the layers of the mesentery and those lying on the loins enormously enlarged forming a large mass filled with dark-coloured and white creamy matter. The veins of the mesentery and those encircling the intestines, purple and congested. There were several large bladders (hydatids I suppose) with thin transparent walls and containing a yellowish fluid lying amongst, and attached to, the agglutinated intestines and viscera, these could be separated from their attachments and taken out entire. Liver covered externally with a layer of lymph which was left attached to it after tearing off the peritoneum, its surface in places of a dark colour and softened in texture, the gall bladder empty, its walls thickened, the cystic duct diminished in calibre, the glands about the hepatic duct enlarged. Spleen studded externally and internally with tubercles of lymph.

*Intestines.* An ulcer at the termination of mucous membrane of Ileum, and another large one at the commencement of the Cœcum, the mucous surface of descending colon in patches red and uneven, a few minute ulcers scattered here and there.

---

### Mr. Wallace's Cases of want of Sagittal Suture in a certain Tribe of Negroes.

27, *Surrey Street, Strand,*

*7th June,* 1836.

Gentlemen,

The following little piece of intelligence you may perhaps think worthy of being presented to your readers.

In December, 1833, His Majesty's Sloop Trinculo, to which I was then attached, while cruizing in the Bight of Biaffra, on the West-coast of Africa, captured two small schooners carrying (both) about 100 slaves, consisting of men, women, and children, the whole of whom we landed immediately after at the island of Fernando Po. The Trinculo did not return to Fernando Po until October 1835, at which time we naturally made inquiry after our old friends the liberated Africans, all of whom, with the exception of those who had died, we found domiciled on the island, having the character of being idle, disobedient, unkind to each other—in short, possessing a host of bad qualities, with scarcely a single good one to redeem them. But the remarkable circumstance to which I wish to call your attention is, that, of those who had died, four had been examined on account of supposed disease in the head, and in all the sagittal suture was wanting. At the first examination the circumstance did not excite any very great degree of surprise, as it was merely supposed to be a *single* deviation from the ordinary process of nature, but when a second, a third, and even a fourth, evinced the same thing, then it not only excited much attention, but produced the very natural conclusion, that in this race of blacks such is the usual cranial conformation. If such is really the case, to say the least of it, it is exceedingly remarkable; and for what purpose Nature should so perform her work is a question which, although interesting, it may not be very easy to determine.

These blacks were principally from that part of the West-coast of Africa lying between the rivers Gatson and Congo, and it has long been noticed that the slaves taken from that quarter are neither so powerfully made, nor in possession of the same degree of intelligence, as the tribes to the northward and westward; but, upon the whole, there is a fair development of skull, and, so far as I have noticed, the mechanism of the head, with the exception alluded to, is perfect.

From Mr. Ballard, surgeon of the establishment at Fernando Po, (the gentleman who kindly furnished me with the information now given,) I received one of the skulls above-mentioned, which, since my arrival in England, I have deposited in the museum at Haslar.

I am, Gentlemen,
Very respectfully your's,
JAMES WALLACE,
*Surg. R.N.*

---

### EASTERN PROVINCIAL MEDICAL ASSOCIATION.

The first annual meeting of this very laudable institution was held lately at Ipswich, where upwards of sixty physicians and surgeons of the neighbouring cities and towns were assembled. Dr. Band, of Ipswich, was called to the chair, and delivered an appropriate address on the occasion. Mr. Crosse, of Norwich, the honorary secretary, read a report. One hundred and sixty subscriptions have been already received—and the number of members exceeds 170. A junction is proposed with the parent association, in order that the Transactions may be published jointly. That thorny subject—the medical management of the poor—was rather warmly discussed, and elicited contrasting sentiments from several of the speakers, viz. Mr. Macintyre, Dr. Cox, Mr. Bree, and Mr. Crosse. Upwards of 50 of the members dined together afterwards, and many appropriate speeches added zest to the wine. We wish the Association every success.

---

### DEATH OF BARRY O'MEARA, ESQ.

This gentleman, so well known as the favourite surgeon of Napoleon, departed this life on the 10th of June, after an illness of ten days, and in the 53d year of his age. He attended a public meeting at the Crown and Anchor the day before his illness commenced, and was then in exuberant health. The next morning he had a rigor, succeeded by violent fever. Having been subject to attacks of pulmonic inflammation, he concluded that this was one of them, and got himself copiously bled. This was an unfortunate mistake. Next morning erysipelas appeared on the face, with a pulse at 140. He sent for Mr. Elmore, his friend, who found him in this state. Dr. Johnson was afterwards called in, and hopes were entertained that life would be preserved. But delirium set in early and continued till the last. The powers of life ebbed —pulse got daily weaker—and although wine was exhibited, the patient sunk on the tenth day of the disease.

The deceased was a very free liver, both as respected food and drink. He was growing corpulent, and experienced paroxysms of gout once or twice a year. He was, therefore, the very worst subject for erysipelas. He never recovered the effects of the copious depletion.

Mr. O'Meara entered the army when very young—we believe when 18 or 19 years of age, and was in Egypt at the battle of Aboukir, when Sir R. Abercromby received his fatal wound. He afterwards served in the Navy, and went with Napoleon to St. Helena.—His life then became public history, and his "Voice from St. Helena" excited universal attention. It also, we believe, procured him a wife—a lady of title and fortune, (the late Lady Leigh,) who settled a thousand a year on her husband. She was advanced in life, and died a few years after their marriage, of hypertrophy of the heart, being attended by Dr. Johnson and others.

After this event, Mr. O'Meara spent his time in the enjoyment of the Society of choice spirits. He had a very large circle of acquaintance in the various clubs of the West End, and being rather an epicure, he wound up the frame of his constitution much too tight—and the consequence was that it gave way at the first onset of a formidable disease.

He was a gentleman of open heart and generous disposition. His political sentiments were strong, and though an enthusiastic admirer of the greatest tyrant of modern times—NAPOLEON—yet he went to the extreme of liberality and reform, of late years. He was the intimate friend of Daniel O'Connel—and in that gentleman's cause, he received the poison which carried him to his grave. At the subscription meeting, in the CROWN and ANCHOR Tavern, Mr. O'Meara stood opposite to an open window, through which a current of cold easterly wind was constantly entering. The room itself was crowded and hot. Here he got the chill, which issued in erysipelas and death! He died at about the same age as his great master and patron, BONAPARTE.

